# Therapeutic resistance and susceptibility is shaped by cooperative multi-compartment tumor adaptation

**DOI:** 10.1038/s41418-019-0310-0

**Published:** 2019-03-01

**Authors:** Jason E. Long, Matthew J. Wongchenko, Dorothee Nickles, Wei-Jen Chung, Bu-er Wang, Johannes Riegler, Ji Li, Qingling Li, Wendy Sandoval, Jeff Eastham-Anderson, Zora Modrusan, Teemu Junttila, Richard A. D. Carano, Oded Foreman, Yibing Yan, Melissa R. Junttila

**Affiliations:** 1 Department of Translational Oncology, Genentech, Inc., 1 DNA Way, South San Francisco, CA 94080 USA; 2 Department of Oncology Biomarker Development, Genentech, Inc., 1 DNA Way, South San Francisco, CA 94080 USA; 3 Department of Bioinformatics and Computational Biology, Genentech, Inc., 1 DNA Way, South San Francisco, CA 94080 USA; 4 Department of Biomedical Imaging, Genentech, Inc., 1 DNA Way, South San Francisco, CA 94080 USA; 5 Department of Microchemistry, Proteomics, & Lipidomics, Genentech, Inc., 1 DNA Way, South San Francisco, CA 94080 USA; 6 Department of Pathology, Genentech, Inc., 1 DNA Way, South San Francisco, CA 94080 USA; 7 Department of Molecular Biology, Genentech, Inc., 1 DNA Way, South San Francisco, CA 94080 USA; 8 Present Address: 23andMe, 899 W Evelyn Ave, Mountain View, CA 94041 USA

**Keywords:** Cancer microenvironment, Cancer genetics, Cancer models, Experimental models of disease, Translational research

## Abstract

Emerging research suggests that multiple tumor compartments can influence treatment responsiveness and relapse, yet the search for therapeutic resistance mechanisms remains largely focused on acquired genomic alterations in cancer cells. Here we show how treatment-induced changes occur in multiple tumor compartments during tumor relapse and can reduce benefit of follow-on therapies. By using serial biopsies, next-generation sequencing, and single-cell transcriptomics, we tracked the evolution of multiple cellular compartments within individual lesions during first-line treatment response, relapse, and second-line therapeutic interventions in an autochthonous model of melanoma. We discovered that although treatment-relapsed tumors remained genetically stable, they converged on a shared resistance phenotype characterized by dramatic changes in tumor cell differentiation state, immune infiltration, and extracellular matrix (ECM) composition. Similar alterations in tumor cell differentiation were also observed in more than half of our treatment-relapsed patient tumors. Tumor cell-state changes were coincident with ECM remodeling and increased tumor stiffness, which alone was sufficient to alter tumor cell fate and reduce treatment responses in melanoma cell lines in vitro. Despite the absence of acquired mutations in the targeted pathway, resistant tumors showed significantly decreased responsiveness to second-line therapy intervention within the same pathway. The ability to preclinically model relapse and refractory settings—while capturing dynamics within and crosstalk between all relevant tumor compartments—provides a unique opportunity to better design and sequence appropriate clinical interventions.

## Materials and methods

### Genetically engineered mouse model (GEMM)

Braf^LSL.V600E^;PTEN^fl/fl^;Tyr.CreER and Braf^LSL.V600E^;PTEN^fl/fl^;Tyr.CreER;Rosa26^LSL.tdTomato^ mice were fully backcrossed (>10 generations) into C57BL/6J mice [1–4]. Licenses were obtained from appropriate institutions.

### Tumor induction

At ~8–12 weeks of age, animals were first anesthetized using continuous 2% Isothesia (Isoflurane, Butler Schien Animal Health; catalog number 1169567762; Dublin, OH). The dorsal skin of the right flank was shaved using an electric shaver, wiped clean, and 1 µL of 5 mM 4-OH tamoxifen (Sigma, catalog number H7904, St. Louis, MO) dissolved in ethanol was applied to the shaved skin to induce tumor formation. After application, the animal was kept under anesthesia for 3 min to allow the ethanol to evaporate, after which they were allowed to recover while monitored on a heating pad. After 4–8 weeks, animals were stratified into treatment cohorts using tumor measurements for in vivo dosing experimentation. They were individually housed to prevent any impact to the induced tumors by another mouse. Equal numbers of male and female animals of ~25 g each were enrolled across dosing groups. Vemurafenib (Zelboraf^®^) was dosed at 50 mg/kg via oral gavage (PO), twice a day (b.i.d.). Cobimetinib (Cotellic^®^) was dosed at 7.5 mg/kg via oral gavage (PO), once a day (q.d.). α-PD-L1 (Clone 9708-6E11) was dosed at 10 mg/kg via intraperitoneal injection, three times a week (t.i.w.). The animals were dosed and monitored according to guidelines from the Institutional Animal Care and Use Committee (IACUC) at Genentech, Inc. (South San Francisco, CA).

### Biopsies

Animals were anesthetized using continuous 2% Isothesia (Isoflurane, Butler Schien Animal Health; catalog number 1169567762; Dublin, OH). The tumor surface and surrounding area was cleaned using 7.5% povidone-iodine and then 75% ethanol. A 3 mm punch biopsy (Miltex, Inc.; catalog number 33-32, York, PA) was used to obtain tumor samples, which were immediately placed into either RNALater (Qiagen, Inc.; catalog number 76104, Valencia, CA) or frozen in liquid nitrogen. After biopsy, the wound was cleaned using a sterile cotton swab and then immediately closed up using 5-0 absorbable sutures (Ethicon, LLC; catalog number J463G, Cincinnati, OH). The animal was allowed to recover while monitored on a heating pad. At the end of treatment, individual solid tumors were collected and trisected for next-generation sequencing analyses and histopathology. All histopathology assessments were blinded.

### Ultrasound imaging

The ultrasound imaging and data analysis has been described in more detail previously [5]. Briefly, mice were anesthetized with sevoflurane (4%), positioned prone on a feedback-controlled heated stage (Visualsonics, Toronto, Canada), fur surrounding tumors was removed using a clipper, and ultrasound gel was applied. To estimate tumor volumes, b-mode images (Siemens Acuson S2000, Munich, Germany) were acquired for axial and sagittal planes covering maximum tumor cross-sections (center frequency 8 MHz, 27% power; 45 µm in plane resolution, 300 µm slice thickness, Field of View (FOV): 3 × 3.8 cm). Tumor boundaries were manually outlined and volumes estimated by fitting an ellipsoid. Two-dimensional Acoustic Radiation Force Impulse (ARFI) imaging (Virtual Touch^TM^ Tissue Imaging Quantification) was performed on axial planes using a clinical transducer (8 MHz). At least four shear wave speed maps were acquired for each imaging plane. Tumor outlines from b-mode images were applied to the average of four corresponding shear wave maps in order to estimate the average shear wave speed for a tumor at a given time point.

### Mass spectroscopy

Tumor tissue were reduced with sodium borohydride, washed with water, dried, hydrolyzed in hydrochloric acid, and transferred to liquid chromatography (LC)-tandem mass spectrometry. A water acetonitrile gradient was run on a reverse-phase column (Agilent Technologies, Santa Clara, CA). The LC was coupled to a mass spectrometer (Applied Biosystems, Foster City, CA) operating under positive ionization mode. Sample analysis was performed in multiple reaction monitoring mode with a dwell time of 0.1 s.

### Survival

Animals were censored for survival in an unblinded manner based on pre-determined morbidity criteria for killing (in consultation with veterinary staff under the IACUC guidelines), which included low body condition scoring (e.g., hunching, belabored breathing, low body temperature), lack of mobility, and > 20% body weight loss from the time of study start or mortality.

### Fluidigm analyses

Tumor RNA was purified using the Qiagen All Prep kit (Qiagen, Inc.; catalog number 80204, Valencia, CA). cDNA was created using Applied Biosystems Reverse Transcription Kit (ABI, Inc.; catalog number 4374966, Waltham, MA). Ten nanograms of tumor RNA was added to 2 µL 10 × Reverse Transcriptase (RT) Buffer, 0.8 µL 100 mM dNTP mix, 2 µL 10 × Random Primers Mix, 1 µL Multiscribe RT, 1 µL of RNase Inhibitor, and 3.2 µL of ddH_2_O, and incubated at 25 °C for 10 min, 37 °C for 120 min, and then 85 °C for 5 min. The resulting cDNA (1.25 µL) was then added to a preamplification reaction containing 2.5 µL of 2 × TaqMan^®^ PreAmp Master Mix (ABI/ThermoFisher Scientific; catalog number 4391128, Waltham, MA) and 1.25 µL of a pool of 96 TaqMan^®^ Assays (ThermoFisher Scientific; catalog number 4331182, Waltham, MA; full list of genes below). This mix was incubated at 95 °C for 10 min and then cycled 14 times at the following conditions: 95 °C for 15 s and 60 °C for 4 min. After the reaction was complete, the mix was diluted with 20 µL of TE Buffer, pH 8.0 (Ambion/ThermoFisher Scientific; catalog number AM9849; Waltham, MA). The resulting amplified cDNA (2.7 µL) was then added to 3 µL of Universal PCR Master Mix (ABI/ThermoFisher Scientific; catalog number 4304437, Waltham, MA) and 0.3 µL of Fluidigm Sample Loading Reagent (Fluidigm; South San Francisco, CA). TaqMan^®^ Gene Expression Assays (3 µL; ThermoFisher Scientific; catalog number 4331182, Waltham, MA; full list of genes below) were added to 3 µL Fluidigm Assay Loading Reagent (Fluidigm; South San Francisco, CA). The resulting mixtures were loaded onto a 96:96 Dynamic Array using a Biomark^TM^ System (Fluidigm; South San Francisco, CA). *C*_t_ values were calculated from the system’s software (Biomark^TM^ Real-time PCR analysis, Fluidigm; South San Francisco, CA). All Raw *C*_t_ values were normalized to one or more of three housekeeping genes, or the whole plate for further analysis.

### Gene signatures

The previously published melanoma differentiation signature contains the following 21 genes: *BACE2*, *CITED1*, *DCT*, *EDNRB*, *GPNMB*, *GPR143*, *KIT*, *MC1R*, *MITF*, *MLANA*, *OCA2*, *PAX3*, *PMEL*, *RARB*, *SLC24A4*, *SLC24A5*, *SLC45A2*, *TRPM1*, *TYR*, *TYRP1*, and *ZEB2* [6]. Our Nanostring gene set contains the following overlapping nine genes with the above melanoma differentiation signature: *BACE2*, *EDNRB*, *GPNMB*, *KIT*, *MITF*, *MLANA*, *PMEL*, *TYRP1*, and *ZEB2*. The mouse melanoma marker gene set contains the following eight genes: *Ednrb*, *S100a1*, *Pmel*, *Lef1*, *Mlana*, *Tyrp1*, *Mc1r*, and *Gpr143*, five of which overlap with our Nanostring gene set and contains the following genes: *EDNRB*, *PMEL*, *LEF1*, *MLANA*, and *TYRP1*. The mitogen-activated protein kinase (MAPK) signature contains the following genes: *Dusp4*, *Dusp6*, *Etv1*, *Etv4*, *Etv5*, *Fosl1*, *Phlda1*, *Spry2*, and *Spry4* [7, 8]. All gene signature values were created by calculating the mean and SD of each gene across all samples. A *z*-score was then obtained for each gene for each sample through the following formula: *z* = (*x* − *µ*)/*σ*, when *x* is the score, *µ* is the mean, and *σ* is the SD. The *z*-score for every gene in the signature for each sample was then added up and divided by the square root of the total number of genes in the signature to give the final plotted value for each sample.

### Whole-exome sequencing

Genomic DNA was extracted from tumor and normal tissues using the Qiagen All Prep nucleic isolation kit (Qiagen, Inc.; catalog number 80204, Valencia, CA) as per the manufacturer’s protocol. Quality and quantity of DNA samples was determined before their processing by exome sequencing. The concentration and the integrity of DNA samples were determined using NanoDrop 8000 (ThermoFisher Scientific, Waltham, MA) and 2200 TapeStation (Agilent Technologies, Santa Clara, CA), respectively. Exome capture was performed using 0.5 µg of genomic DNA and SureSelectXT Mouse All Exon kit (50 Mb) according to the manufacturer’s protocol (Agilent Technologies, Santa Clara, CA). Fragment size distribution of post-capture amplified libraries was determined with 2200 TapeStation using high-sensitivity D1000 screen tape (Agilent Technologies, Santa Clara, CA). Concentration of the libraries was measured by Qubit (ThermoFisher Scientific, Waltham, MA). Exome capture libraries were sequenced on HiSeq2500 (Illumina, San Diego, CA) to generate paired-end 75 base reads.

### RNA sequencing

Total RNA was extracted from cells using the Qiagen All Prep nucleic isolation kit (Qiagen, Inc.; catalog number 80204, Valencia, CA) as per the manufacturer’s protocol, including the on-column DNase digestion. Quality control of samples was done to determine RNA quantity and quality before their processing by RNA sequencing (RNA-seq). The concentration and the integrity of total RNA samples were determined using NanoDrop 8000 (ThermoFisher Scientific, Waltham, MA) and 2200 TapeStation (Agilent Technologies, Santa Clara, CA), respectively. One microgram of total RNA was used as an input material for library preparation using TruSeq RNA Sample Preparation Kit v2 (Illumina, San Diego, CA). Library size was confirmed using the 2200 TapeStation and high-sensitivity D1K screen tape (Agilent Technologies, Santa Clara, CA), and their concentration was determined by quantitative PCR-based method using Library quantification kit (Kapa Biosystems, Wilmington, MA). The libraries were multiplexed and then sequenced on HiSeq2500 (Illumina, San Diego, CA) to generate 30 M of single-end 50 base pair reads.

### Analysis of whole-exome and RNA sequences

Whole-exome and RNA sequences from initial biopsy (IB) tumors, progressed biopsy tumors (V_Pr_), and matched normal genetically engineered mouse models (GEMM) were trimmed to 75 bp and filtered for sequencing quality and ribosomal RNA. Reads for which 30% or more of the nucleotides had a Phred quality score of 23 or lower were discarded. The remaining reads were aligned to the mouse genome using the Genomic Short-read Nucleotide Alignment Program (GSNAP) [9], with default settings and the following parameters: -M 2 -n 10 -B 2 -i 1 -N 1 -w 200000 -E 1 --pairmax-rna = 200000 --clip-overlap. Further trimming was not performed. Multimapping reads were discarded. PCR duplications were filtered using PicardTools. We used established gene models from the National Center for Biotechnology Information (NCBI) database and extended those with internal Genomic Mapping and Alignment Program for mRNA and EST sequences (GMAP) alignments for additional genes not in NCBI database. We used GATK (Version 3.5) to measure the sequencing depth and the coverage in targeted regions of whole-exome sequencing. For whole-exome sequencing, somatic single-nucleotide variants and insertion/deletions were called using Strelka 1.0.4 with its default settings [10]. High-confident variants were annotated by Ensembl Variant Effect Predictor (version 77) [11] and filtered with dbSNP 138, RepeatMasker 4.0.5 (http://www.repeatmasker.org), and variants obtained from normal tails of C57BL/6J mice collected in multiple internal projects. We considered somatic variants as high-confident variants that passed our filtering and were only observed in matched second biopsy tumors, not in the matched normal samples. Protein-altering mutations include nonsynonymous mutations, gain/loss of stop codon, insertion/deletion, frameshift mutations, and mutations at splicing donor and acceptor sites. Human cancer-related genes were obtained from ref. [12]. To confirm expression of somatic variants, we used the VariantTools and gmapR packages in Bioconductor and counted the number of RNA-seq reads carrying the same somatic variants in the BAM files of matched samples that align to the genomic coordinates of the variants. We manually examined PAMs using Integrative Genomics Viewer. We also aligned exome reads containing recurrent variants to the mouse genomic and transcripts database using BLASTN, and included alignment to alternate assemblies, such as Mm_Celera in Extended Dataset 1. Gene expression levels were quantified from RNA-seq as reads per kilobase of exon model per million mapped reads normalized by size factor, referred to as RPKM, and defined as the number of reads aligning to a gene in a sample, divided by the total number of uniquely mapped reads for that sample × gene length × size factor.

### Detection of copy number aberrations

We inferred the copy number (CN) landscape of each tumor from its exome sequence and the exome sequence of its matched normal, using Control-FREEC with the default exome-seq settings (window size 500 bases and step 250 bases) [13]. CN per gene was obtained by averaging the coverage of segments containing the gene of interest and was used to screen for CN gain (2.8 ≤ CN < 4), amplification (CN ≥ 4), loss (1 < CN ≤ 1.4), and deletion (CN ≤ 1). Regions of focal amplification/deletion are defined as regions <5 Mb. Whole chromosome gain is defined as gain/amplification of at least 80% of genes per chromosome. Prevalence of gene amplification, gain, loss, or deletion was calculated as the ratio of number of tumors with the alteration to the total number of tumors with available CN status for that gene.

### Nanostring RNA analysis of patient tumors

BRIM2 (NCT00949702) was a single-arm phase 2 study in which patients with BRAF^V600^-mutant melanoma received vemurafenib treatment. Patient consent was obtained for exploratory research conducted on all tissues. mRNA was prepared from formalin-fixed, paraffin-embedded sections of tumor tissue and gene expression was measured using Nanostring (NanoString Technologies, Seattle, WA). Data were normalized to the geometric mean of all 800 genes profiled. The effect on baseline expression was determined using a Cox proportional hazards model. For tissues from melanoma biopsies, sections were stained with hematoxylin and eosin. RNA was isolated using the High Pure RNA Paraffin Kit (Roche; catalog number 03270289001, Basel, Switzerland).

### Immunohistochemistry

Formalin-fixed, paraffin-embedded sections of murine melanoma were cut at 5 µm. Sections were then deparaffinized and subjected to antigen retrieval using Target Retrieval Solution (Agilent Technologies, catalog number S1700, Santa Clara, CA) at 98 °C for 10 min and allowed to cool at room temperature for 30 min. Melanoma sections were then blocked for 1 h in phosphate-buffered saline (PBS) containing 0.1% Triton X-100, 3% bovine serum albumin and 5% normal donkey serum (Jackson Immuno Research Labs, catalog number NC9624464, West Grove, PA). Thereafter, they were incubated overnight at 4 °C in the same buffer with the following primary antibodies: Anti-tdTomato (1:500) (Biorbyt, catalog number ORB182397, San Francisco, CA) and anti-phospho ERK (1:250) (Cell Signaling Technology, catalog number 4370, Danvers, MA). After three PBS washes, cultures were incubated for 1 h at room temperature with the following secondary antibodies: Alexa488-conjugated anti-rabbit (1:400) and Alexa594-conjugated anti-goat (1:400) (ThermoFisher Scientific, Waltham, MA). Slides were counterstained with 4,6-diamidino-2-phenylindole, dihydrochloride (ThermoFisher Scientific, catalog number D1306, Waltham, MA) and mounted using ProLong Gold Antifade mountant (ThermoFisher Scientific, Waltham, MA). Fluorescent photomicrographs were taken on a Zeiss microscope (Carl Zeiss Microscopy, Thornwood, NY). All picture acquisitions were as individual tiff files and composite images were made in Adobe Photoshop CC (Adobe Systems, Inc., San Jose, CA).

### Cell culture

Briefly, polyacrylamide coverslips were coated as described in refs. [14, 15], substituting fibronectin at 20 mg/mL (Sigma, catalog number F1141, St. Louis, MO) for collagen. Approximately 12 µL of matrix stiffness solution (0.2 kPa—10 mM HEPES 424.7 µL, 40% Polyacrylamide 37.5 µL, 2% Bisacrylamide 7.5 µL, APS 2.5 µL, TEMED 0.25 µL; 3 kPa—10 mM HEPES 403 µL, 40% Polyacrylamide 68.6 µL, 2% Bisacrylamide 22.48 µL, APS 2.5 µL, TEMED 0.25 µL; 12 kPa—10 mM HEPES 358 µL, 40% Polyacrylamide 94.4 µL, 2% Bisacrylamide 40 µL, APS 2.5 µL, TEMED 0.25 µL) was covered with a cover glass and left for 1 h at room temperature to set the gel. After removing the cover glass, the gel was coated with 150 mL of Sulfo-SANPAH Photoreactive Crosslinker (Pierce/ThermoFisher, Waltham, MA) and activated by UV illumination for 10 min, followed by fibronectin coating. A375 (gCell number CL584727, Genentech, South San Francisco, CA), Colo829 (gCell number CL131105, Genentech, South San Francisco, CA), MelNBR1 (cell line generated from an induced tumor from the Braf^LSL.V600E^;PTEN^fl/fl^;Tyr.CreER GEMM), and WM266-4 (gCell number CL586497, Genentech, South San Francisco, CA) cells in Dulbecco’s modified Eagle’s medium were seeded onto the gels, cultured for 72 h. Cells were processed for analyses as described in the Fluidigm Analyses section above.

### Generation of single-cell RNA-seq data

Processing of melanoma tumors for flow cytometry was done by cutting the tumors into small pieces of 2–4 mm with a razor blade. Two milliliters of ice-cold Digestion Buffer (5 mg/mL collagenase, type I; 5 mg/mL collagenase, type IV) in Hank’s balanced salt solution (HBBS) was then added to the tumor pieces in 15 mL tubes. Samples placed at 37 °C for 20 min in the dark. Digestion was stopped by adding 2 mL of ice-cold Washing Buffer (20% FCS in HBBS). Digest was pelleted at 1500 r.p.m. for 5 min at 4 °C. The pellet was washed once with 1 × PBS and then incubated in 2 mL of 0.25% trypsin in HBBS at 37 °C for 5 min in the dark. Reaction was stopped by adding 2 mL of ice-cold Washing Buffer and then digest was pelleted at 1500 r.p.m. for 5 min at 4 °C. Pellet was resuspended in 5 mL of ice-cold fluorescence-activated cell sorting (FACS) Buffer (2% fetal bovine serum, 2 mM EDTA in 1 × PBS). Digested tumors were passed through a 70 μm strainer. The cell strainer was washed with 5 mL of FACS Buffer and samples centrifuged at 1500 r.p.m. for 5 min at 4 °C. Pellet was washed once with 5 mL of FACS Buffer and pelleted again at 1500 r.p.m. for 5 min at 4 °C. Cells were resuspended in 1 mL of FACS Buffer + propidium iodide.

Samples were processed for single-cell RNA-seq (scRNAseq) using the Chromium Single Cell 3’ Library and Gel bead kit v2, following the manufacturer’s manual (10 × Genomics, San Francisco, CA). Cell density and viability of the single-cell suspensions were determined by Vi-CELL XR cell counter (Beckman Coulter). All of the processed samples had very high percentage of viable cells. Cell density was used to impute the volume of single-cell suspension needed in the RT master mix, aiming to achieve ~6000 cells per sample. cDNAs and libraries were prepared following the manufacturer’s manual (10 × Genomics, San Francisco, CA). Libraries were profiled by Bioanalyzer High Sensitivity DNA kit (Agilent Technologies, Santa Clara, CA) and quantified using Kapa Library Quantification Kit (Kapa Biosystems, Wilmington, MA). Each library was sequenced in one lane of HiSeq4000 (Illumina, San Diego, CA) following the manufacturer’s sequencing specification (10 × Genomics, San Francisco, CA).

### Processing of scRNAseq data

The number of reads for all ~700k possible cell barcodes were tallied and data demultiplexed for cell barcodes represented by at least 10k reads. Transcript reads were aligned to the reference genome GRCm38 using GSNAP version “2013-10-10” (parameters: ‘-M 2 -n 10 -B 2 -i 1 -N 1 -w 200000 -E 1); only uniquely mapping reads were considered. The number of transcripts per gene was inferred based on the number of unique molecular identifiers (UMIs) per gene (for reads overlapping exons in sense orientation) allowing for one mismatch between UMI sequences to account for errors in sequencing or PCR amplification. After excluding cells with <300 UMIs and removing non-expressed features, data were further processed using the Seurat R package version 2.2.0) [16]. Subsequent to normalization using the “LogNormalize” setting, data were scaled based on the total number of UMIs per cell. Then a principle component (PC) analysis was run on the most variably expressed genes and the first 30 PCs were used to perform *t*-distributed statistical neighbor embedding analysis and density clustering. All scRNAseq data has been deposited into the Gene Expression Omnibus (GEO) repository at NCBI. It can be found using the accession number GSE126714.

### Assignment of cluster identities in scRNAseq data

As quality control, we visualized the percentage of total reads that aligned to the mitochondrial genome to identify cells undergoing cell stress/death [17] as well as relative expression of cell cycle genes to identify clusters where the cells’ transcriptomes were more indicative of cell cycle state than cell identity. Clusters with a high fraction of mitochondria rich or actively dividing cells were excluded from analysis. We then used the FeaturePlot function to highlight expression of known marker genes for the different cell types that were expected to be present in the samples: transgene (tGene) expression marking tumor cells, *Fap* and collagens marking non-immune stromal cells, hemoglobin marking red blood cells, *Pecam1* marking endothelial cells, as well as different immune cell markers as visualized in Extended Data Fig. 5a). In parallel, using Seurat’s “FindAllMarkers” function, marker genes for each respective cluster were identified in a hypothesis-free manner and reviewed.

### Visualization of scRNAseq data

For the heatmap in Extended Data Fig. 5a, expression of select marker genes was averaged across all cells in each cluster using Seurat’s “AverageExpression” function. The violin plots show the kernel probability density of the data. The white point indicates the mean expression. The thick black lines extend to the 25th and 75th percentiles of the data (hinges), whereas the thin lines show the largest or smallest observation that falls within a distance of 1.5 times the length of the thick black line from the nearest hinge. The MAPK gene signature score was calculated using Seurat’s “AddModuleScore” function, based on the following MAPK target genes: *Dusp4*, *Dusp6*, *Etv1*, *Etv4*, *Etv5*, *Fosl1*, *Phlda1*, *Spry2*, and *Spry4*.

### Statistics

Statistical analyses are indicated in Figure legends. Data are presented as the mean ± SD. GraphPad Prism 7 software (GraphPad Software, La Jolla, CA) was used to conduct the statistical analysis of all data. Multiple comparisons were performed using an ordinary one-way analysis of variance with Tukey’s multiple comparison test. Survival comparisons were made using the log-rank (Mantel–Cox) test. Paired *t*-tests were used to determine two-tailed significance to compare results. A *P*-value of <0.05 was considered statistically significant.

### Study approval

All individuals participating in animal care and use are required to undergo training by the institution’s veterinary staff. Any procedures, including handling, dosing, and sample collection mandates training and validation of proficiency under the direction of the veterinary staff before performing procedures in experimental in vivo studies. All animals were dosed and monitored according to guidelines from the IACUC on study protocols approved by the Laboratory Animal Resource Committee (LARC) at Genentech, Inc. All patients who participated in the clinical trials provided written, informed consent.

## Results

**Fig. 1:**
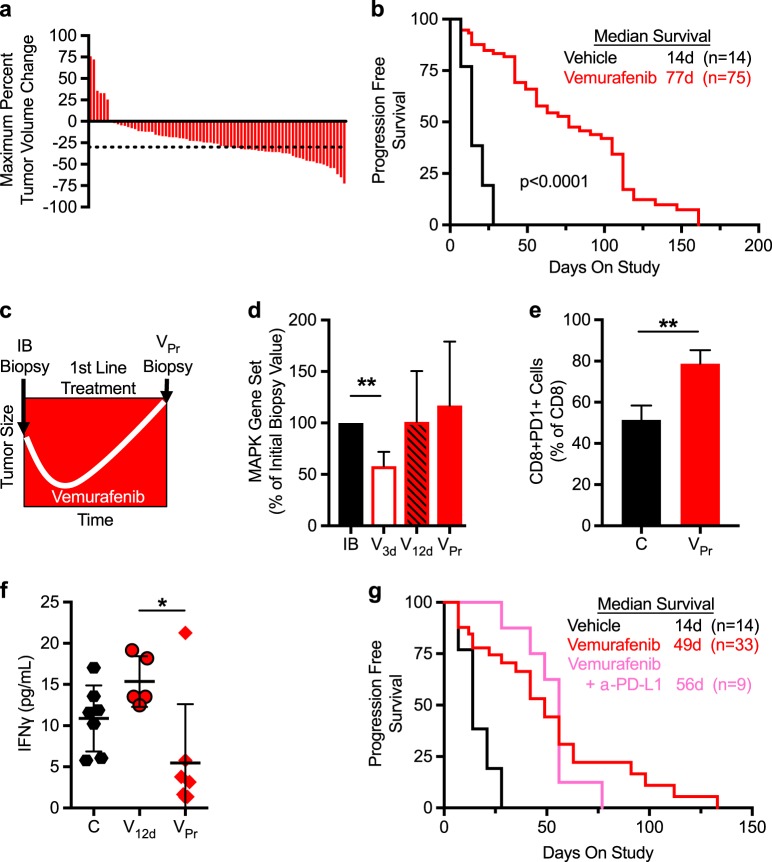
Braf mutant tumors relapse on vemurafenib with evidence of MAPK pathway re-activation and immune modulation. **a** Maximum percent tumor volume change in 75 animals treated with vemurafenib, twice a day via oral gavage at 50 mg/kg. Dotted line indicates  ~30% regression. **b** Progression-free survival plot of vehicle and vemurafenib-treated animals. Animals were classified as progressed when their tumor size exceeded 30% of their initial biopsy value. **c** Schematic representation of tumor growth curve over time while on vemurafenib, showing initial biopsy (IB) and progressed biopsy (V_Pr_) sample time points. **d** Sum of normalized 2^−dCt^ values of MAPK target genes (*Dusp4*, *Dusp6*, *Etv1*, *Etv4*, *Etv5*, *Fosl1*, *Phlda1*, *Spry2*, and *Spry4*) plotted as percent of IB for V_3d_(*n* = 5), V_12d_(*n* = 6), and V_Pr_(*n* = 75) samples. **e** FACS analysis of CD8 + PD1 + cells, plotted as a percent of total CD8 + cells for C(*n* = 3) and V_Pr_(*n* = 3) samples. **f** Luminex analysis of IFNγ expression from C(*n* = 7), V_12d_(*n* = 5), and V_Pr_(*n* = 7) tumors. **g** Progression-free survival plot of control vehicle (*n* = 14), vemurafenib (*n* = 33), and vemurafenib + α-PD-L1 (*n* = 9)-treated animals. Animals were classified as progressed when their tumor burden reached 30% above their initial biopsy value. Data are plotted as the mean ± SD for **d**–**f**. *P*-values in **a** by log-rank (Mantel–Cox) test. *p < 0.05, **p < 0.005 by *t*-test in **e**, **f**. C: control vehicle-treated samples; IB: initial biopsy; V_3d_: 3 day vemurafenib-treated samples; V_12d_: 12-day vemurafenib-treated samples; V_Pr_: vemurafenib-progressed samples

BRAF mutant melanoma is an indication where clinical implementation of small molecule inhibitors targeting BRAF proved transformative [18, 19]; however, the majority of patients progress within the first year of treatment [20, 21]. Although most patients progress with evidence of MAPK pathway re-activation, this is not always attributable to acquired mutations within the pathway [22–26]. To experimentally explore vemurafenib response and relapse, we employed an autochthonous melanoma mouse model, Braf^V600E^;PTEN^fl/fl^;Tyr:CreER (hereafter Braf^V600E^;PTEN) [3]. Vemurafenib treatment regressed over 90% of the tumors and significantly improved progression-free survival (PFS) by >60 days (Fig. 1a, b). Time-to-treatment relapse was not associated with baseline *Mitf* expression levels nor *Mitf/Axl* ratios (Extended Data Fig. 1a, b) [27–29]. Despite continuous vemurafenib treatment, all tumors eventually relapse (Fig. 1b).

To interrogate the mechanism of therapeutic relapse, serial biopsies were performed on the same lesion throughout treatment (IB and vemurafenib-progressed, V_Pr_; Fig. 1c). Consistent with clinical evidence [30], V_Pr_ samples no longer showed significant reduction in MAPK signaling (Fig. 1d, Extended Data Fig. 1d). BRAF inhibitors exhibit mechanistically distinct functions depending on BRAF mutational status; although inhibition of MAPK signaling occurs in BRAF mutant cells, BRAF wild-type cells are primed for activation [31, 32]. To enable facile differentiation of tumor cells from stromal cells, Braf^V600E^;PTEN mice were interbred with a conditional reporter strain (Rosa26.LSL.tdTomato) [4]. Analysis of V_Pr_ tumors from these animals indicated that both positive and negative tdTomato cells had increased pERK at relapse (Extended Data Fig. 1e), consistent with previous work showing that chronic treatment with vemurafenib can lead to MAPK activation within stromal cells [15, 31]. Therefore, both tumor and stromal cells contribute to overall MAPK signaling at vemurafenib relapse.

To determine whether treatment progression is characterized by acquired genomic alterations, 17 matched biopsies (IB and matched V_Pr_) were subjected to RNA and exome sequencing (Extended Data Table 1). No evidence of acquired protein-altering mutations (PAMs) in oncogenes or tumor suppressors in V_Pr_ tumors was discovered and no recurrent PAMs were found in any gene where expression could be verified by RNA-seq (Supplementary Dataset 1). In addition, no significant or recurrent copy number alterations (CNA) were observed and notably, none in BRAF exons [33, 34] (Extended Data Fig. 1e,f). Therefore, vemurafenib progression is not associated with acquired genomic alterations in this model system. Consistent with this finding, a recent report using a distinct BRAF mutant model found vemurafenib relapse in the absence of acquired mutations unless rendered genetically unstable through telomerase inactivation [35].

Further, although individual matched IB and V_Pr_ pairs demonstrated expression changes of receptor tyrosine kinases (RTKs) previously implicated in mitogen-activated protein kinase inhibitor (MAPKi) progressing patients [36], no statistically significant changes were observed in *Axl*, *Pdgfr*, *Egfr*, *Igfr*, *Met*, or *Fgfr*, eliminating identification of a common upregulated RTK that mediates resistance (Extended Data Fig. 1h). Interestingly, Gene Set Enrichment Analysis revealed a significant negative enrichment for T-cell activation, inflammatory, and cytokine production signatures in V_Pr_ lesions (Extended Data Fig. 2a). Evaluation of the immune contexture found V_Pr_ tumors harboring a significant increase in myelosuppressive CD11b + /Gr1 Hi + cells [37], increased CD8 + cells, as well as CD8 + PD1 + cells (Fig. 1e, Extended Data Fig. 2b, c). Moreover, PD-L1 transcript expression increased in ~50% of V_Pr_ samples (8/17 tumors; Extended Data Fig. 2d).

Although acute 12-day vemurafenib treatment (V_12d_) displayed increasing amounts of CD8 + cells, tumor necrosis factor (TNF), and interferon-γ (IFNγ) protein expression, the only effect maintained at V_Pr_ was increased CD8 + and CD8 + PD1 + cells (Fig. 1e, f, Extended Data Fig. 2e–g). Despite increased expression of checkpoint proteins PD1/PD-L1 in V_Pr_ samples, combination vemurafenib and α-PD-L1 therapy provided no benefit in PFS relative to vemurafenib (Fig. 1g), suggesting that the PD1/PD-L1 axis is not functionally contributing to vemurafenib progression in this model.

**Fig. 2:**
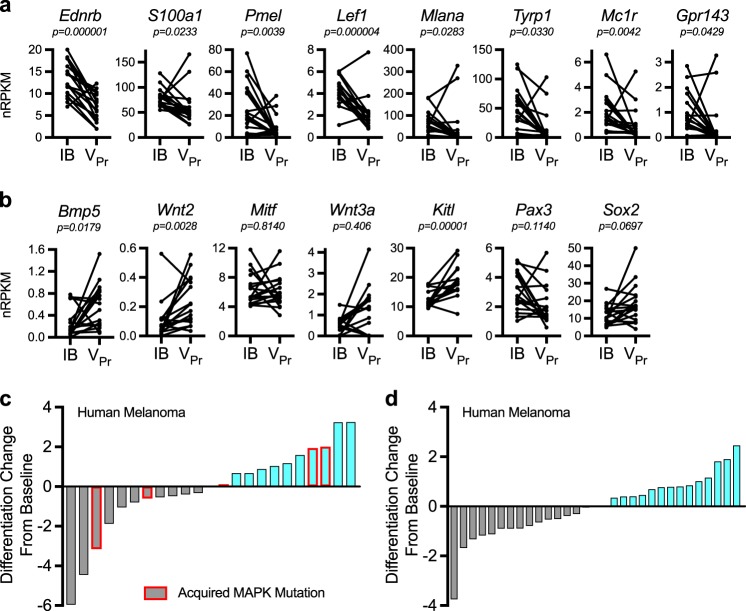
Melanocyte cell-fate gene expression characterizes vemurafenib relapse in murine and human tumors. **a**, **b** Normalized RNA-seq RPKM values plotted for matched biopsies for mature melanocyte markers (**a**, *n* = 17) or neural crest-like markers (**b**, *n* = 17). **c** Differentiation change from baseline score of melanoma differentiation genes (*BACE2*, *EDNRB*, *GPNMB*, *KIT*, *MITF*, *MLANA*, *PMEL*, *TYRP1*, *ZEB2*) derived from ref. [6] in matched pre-treatment and progressed biopsies from melanoma patient samples (*n* = 23 pairs) from BRIM clinical trials [41] and **d** from melanoma patient samples (*n* = 31 pairs) from ref. [21]. Bars outlined in red are patients whose tumors acquired a MAPK pathway mutation at time of vemurafenib progression. Data are plotted as direct RPKM values in **a**, **b**. *p*-values determined by *t*-test in **a**, **b**. IB: initial biopsy; V_Pr_: vemurafenib-progressed samples

Interestingly, genes whose expression characterizes mature terminal melanocyte differentiation were among the most differentially expressed genes between matched IB/V_Pr_ pairs (Fig. 2a), despite heterogeneous baseline expression. In contrast, genes indicative of pre-melanocytic lineage [38–40] displayed mixed expression patterns with statistically significant changes observed in neural crest associated genes *Bmp5*, *Kitl*, and *Wnt2* (Fig. 2b). Of note, transcriptional changes in *Mitf* were not prominent (1/17 up 2-fold), but, when present, terminal melanocyte differentiation genes were concurrently altered (Extended Data Fig. 3c). Using a signature derived from single-cell analysis of transformed melanocytes in the *Braf*^*V600E*^; *PTEN* model [6], we confirmed decreased differentiation in V_Pr_ tumors (Extended Data Fig. 3a, b), indicating that chronic vemurafenib treatment reduces melanocyte marker gene expression heterogeneity either by directly impacting tumor cells or by enriching for a pre-existing cell state within the tumor.

Early time points demonstrated evidence of inflammation, a feature previously associated with melanoma cell dedifferentiation [41]. However, at the time of frank tumor relapse, over 88% (15/17) of tumors were dedifferentiated with no evidence of observable inflammation, indicating that inflammation is not required to maintain this phenotype (Fig. 1f, Extended Data Fig. 2g). Moreover, treatment withdrawal from the V_Pr_ tumors did not induce re-expression of melanocyte marker genes (data not shown), suggesting irreversibility within this timeframe.

We followed up on the cell-state conversion phenotype using matched pretreatment and post-progression melanoma patient biopsy samples (*n* = 23 pairs) [42], as this phenomenon was suggested to accompany clinical MAPKi progression [43]. Transcriptome analysis revealed that 57% (13/23) of patients demonstrated altered differentiation status at the time of vemurafenib progression (Fig. 2c). This observation was confirmed in an independent patient cohort where 52% (16/31) of BRAF inhibitor (BRAFi)-progressed patient biopsies showed a similar decrease in gene expression (Fig. 2d) [22]. In both clinical data sets, our murine-derived gene set also confirmed melanocyte differentiation changes with 43% and 58% of patients showing a decrease, similar to the 55% observed using a previously published murine signature [6] (Extended Data Fig. 4a–c). Interestingly, only five patients in our clinical dataset acquired a MAPK pathway mutation at vemurafenib progression (22%); these MAPK mutations were not mutually exclusive to that of decreased terminal melanocyte differentiation (Fig. 2c). In summary, the acquisition of the dedifferentiated cell state at the time of vemurafenib relapse is occurring at least as frequently as MAPK pathway mutations in *BRAF* mutant melanoma.

**Fig. 3:**
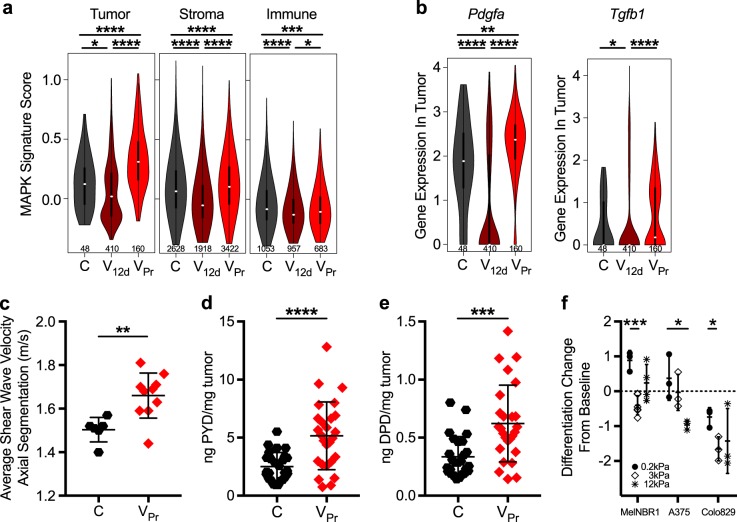
Vemurafenib-associated matrix remodeling impacts melanoma cell-state changes. **a** Single-cell RNA-seq violin plot showing expression of the MAPK gene signature in tumor, non-immune stromal, and immune cells. In all three cellular compartments, MAPK activity is significantly repressed in the V_12d_ tumor cells but re-activated in the V_Pr_ tumor cells. Number of cells per group is given below the violin plots. **b** Single-cell RNA-seq violin plots showing the expression of *Pdgfa* and *Tgfb1* in tumor cells split by treatment. Expression of stroma-recruiting factors is increased in untreated and progressing tumor cells as compared with regressing tumor cells. **c** Average shear wave velocity measurement plotted for C (*n* = 6) and V_Pr_ (*n* = 10) tumors, reflecting the stiffness of tumors. **d**, **e** Mass spectrometry of pyridinoline (PYD) and deoxypyridinoline (DPD) from C (*n* = 26) and V_Pr_ (*n* = 26) tumors. **f** Differential impact of stiffness on the melanoma differentiation signature in three *Braf*^*mut*^ cell lines. Differentiation change from baseline score was derived using the expression of the eight genes from Fig. 2a (*Ednrb*, *S100a1*, *Pmel*, *Lef1*, *Mlana*, *Tyrp1*, *Mc1r*, *Gpr143*). Human melanoma-derived cell lines, A375 and Colo829, and murine Braf^V600E^; PTEN melanoma-derived cell line, MelNBR1, were used. MelNBR1 expressed only three of the eight genes (*Pmel*, *Lef1*, *Mc1r*). *p < 0.05, ***p < 0.001, ****p < 0.0001 by Wilcoxon rank-sum test in **a**; data are plotted as the mean ± SD for **c**–**f**. *p < 0.05, **p < 0.005, ***p < 0.0005, ****p < 0.00005 by *t*-test in **c**–**f**. C: control vehicle treated samples; IB: initial biopsy; V_12d_: 12-day vemurafenib treated samples; V_Pr_: vemurafenib-progressed samples

Fidelity between the resistance phenotype observed in the murine model and that of >50% of patient samples from our own unpublished, as well as an independent clinical cohort, provided rationale for studying such a prevalent clinical outcome and, moreover, for applying this particular model system to elucidate mechanistic features of this type of therapeutic resistance. In order to assess the chronology of changes in individual tumor compartments during treatment relapse, we employed scRNAseq on naive, V_12d_ and V_Pr_ tumors. Indeed, the transient nature of MAPK pathway suppression was confirmed; re-activation of MAPK signaling was observed in tumor cells, but also apparent in non-immune stromal cells (Fig. 3a). Further, single-cell transcriptomics confirmed broad qualitative changes in immune cell infiltrate; most notably, regressing tumors showed the highest infiltration by NK/T cells, with a relative decrease in monocytes/macrophages (Extended Data Fig. 5b). The significantly elevated expression of stromal cell-recruiting factors *Pdgfa* and *Tgfb1* in tumor cells, as well as a strikingly high non-immune stromal cell/tumor cell ratio in naive and V_Pr_ tumors as compared with the regressing tumor (55 and 21 vs. 5, respectively; Fig. 3b, Extended Data Fig. 5c), indicate dynamic changes within the stromal compartment during treatment relapse.

Histological examination of matched patient biopsies showed that 50% (10/20) displayed increased thickness and abundance of haphazardly organized collagen fibers in the extracellular matrix (ECM), consistent with previous reports [15]. Paucicellular dense connective tissue surrounded blood vessels and separated neoplastic cells at the time of vemurafenib progression (Extended Data Fig. 6a). Moreover, paired transcriptomics revealed decreased melanogenesis-related gene expression in all patient tumors with concomitant evidence of ECM changes at vemurafenib relapse (Fig. 2c).

As tumor stiffness has been previously implicated in drug-resistant niche development [15, 44], we sought to further characterize the treatment-induced matrix changes in greater detail. Using ARFI imaging [5], we discovered a significant increase in tumor stiffness in vivo in V_Pr_ tumors (Fig. 3c). Furthermore, both mature pyridinoline and deoxypyridinoline collagen crosslinks were significantly increased in V_Pr_, as determined by mass spectrometry (Fig. 3d, e; Extended Data Fig. 6b, c). Indeed, non-immune stromal cells in the V_Pr_ tumor expressed significantly more *Tgfb1* and *Tgm2* (encoding a collagen crosslinking enzyme), and scored high for a fibroblast transforming growth factor-β response signature (PMID: 29443960), which included genes related to myofibroblast function (Extended Data Fig. 5c). Together, these data suggest an association between matrix remodeling and the differentiation status of the melanoma tumor cells in both the human and murine setting.

Having observed that matrix stiffness and collagen crosslinking are significantly greater in vemurafenib-progressing tumors in vivo, and that 88% of V_Pr_ tumors display decreased differentiation, we sought to directly interrogate this relationship by assessing whether matrix stiffness was sufficient to induce melanocyte gene expression changes. In response to increasing matrix rigidity, human and murine melanoma-derived cell lines exhibited significant decreases in melanocyte marker gene expression at stiffness levels ≥3 kPa (Fig. 3f, Extended Data Fig. 6d), indicating that alterations of ECM stiffness can directly impact melanocyte marker gene expression independently from inflammation and vemurafenib exposure. To understand whether these ex vivo matrix-induced cell-fate changes could impact therapeutic responsiveness, we subjected matrix-converted cells to BRAFi- and MEK inhibitor (MEKi)-targeted therapies, and observed significant protection imparted by increased matrix stiffness (Extended Data Fig. 6g, h). Together, this data indicates that matrix evolution can direct tumor cell-fate changes and negatively impact treatment susceptibility.

Next, we sought to test the functional consequences of the progressed tumor state—characterized by dedifferentiation, altered immune contexture, MAPK pathway re-activation, and increased stiffness—by assessing second-line MEKi treatment intervention responses. V_Pr_ tumor responses to second-line cobimetinib treatment were significantly impaired relative to vemurafenib-naive tumors (PFS 77–252 days, respectively), despite evidence of significant MAPK pathway suppression (Extended Data Fig. 7a, b) [45, 46]. This recapitulates differences observed in clinical responses to combination vemurafenib and cobimetinib treatment between V_Pr_ melanoma vs. treatment-naive (PFS 2.8 vs. 13.8 months) [47]. Notably, although vemurafenib (BRAFi) failed to provoke acquired genomic at relapse, exome sequencing of matched pretreatment and first-line MEKi progressors demonstrated acquired genetic alterations mostly in the form of CNA (Extended Data Fig. 7c). Thus, mechanisms of therapeutic resistance in this model are impacted by the distinct selective pressure, i.e., are both treatment-dependent and specific. Together, our findings show that prior lines of therapy can significantly impair treatment response relative to treatment-naive settings, both in a preclinical and clinical context, emphasizing the importance of considering known mechanisms of resistance in treatment decisions and sequencing.

**Fig. 4:**
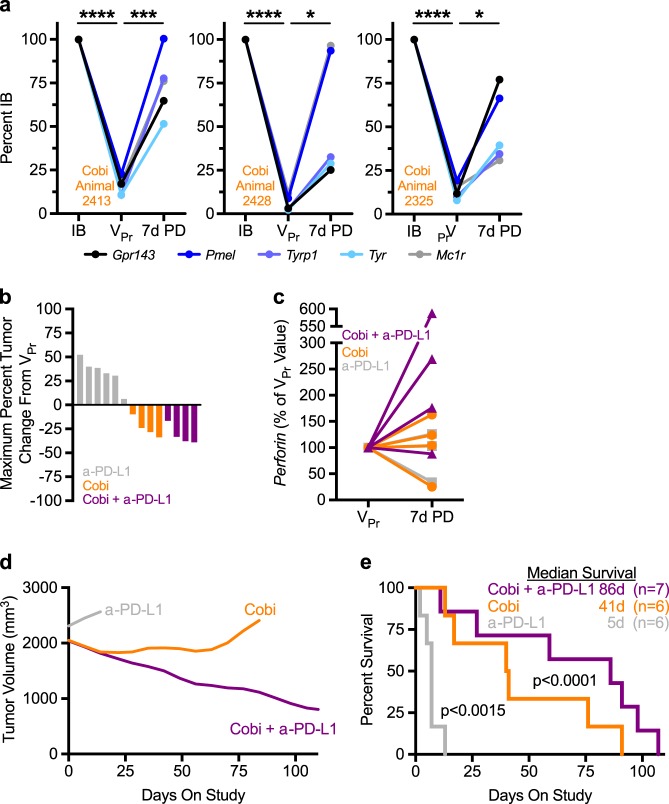
Second-line MAPK inhibition intervention reverts vemurafenib-progressed cell state and increases survival. **a** Matched melanoma differentiation marker gene expression from initial biopsy (IB), vemurafenib-progressed (V_Pr_), and day 7 post second-line crossover cobimetinib-treated (7d PD) tumors plotted as a percent of IB. **b** Maximum percent tumor volume change from V_Pr_ by treatment. **c** *Perforin* transcript plotted as a percent of V_Pr_ plotted for three different day 7 post second-line crossover treated (P (gray, *n* = 3), Co (orange, *n* = 4), and CP (purple, *n* = 4)) tumors. **d** Fourth-order smoothed tumor volume plot for second-line treatments (see Extended Data Figure 7a for raw data). **e** Kaplan–Meier plot of animals by treatment group with median survival. Notably, no animals were killed in the combination treatment group due to tumor regrowth. This was not the case for the single agent α-PD-L1 (100% of animals) and cobimetinib (50% of animals) groups. *p < 0.05, **p < 0.005, ***p < 0.0005, ****p < 0.00005 by *t*-test in **a**. *P*-values in **e** by log-rank (Mantel–Cox) test. 7d PD: vemurafenib-progressed samples that are treated for 7 days with various second-line treatments; Co: vemurafenib-progressed cobimetinib-treated samples; CP: vemurafenib-progressed cobimetinib- and α-PD-L1-treated samples; IB: initial biopsy; P: vemurafenib-progressed α-PD-L1-treated samples; V_Pr_: vemurafenib-progressed samples

Given that chronic vemurafenib treatment altered melanoma dependency and responsiveness to second-line intervention, we sought to more fully explore the pharmacodynamic impact of MEKi on vemurafenib-relapsed tumors. Acute MEKi treatment of V_Pr_ tumors resulted in significant immune contexture changes, including decreased myeloid-derived suppressor cell occupancy, increased CD8 infiltration, and major histocompatibility complex-I (MHC-I) expression (Extended Data Fig. 7d–f), features previously reported in response to MEKi in other tumor types and con [48, 49]. In addition, MEKi treatment lead to significant re-expression of melanocyte marker genes within tumors, which are also known tumor-associated antigens (Fig. 4a). Taken together, MEK inhibition of the relapsed tumors engenders pleiotropic effects on both tumor and immune cells that could potentially be leveraged using rationale combination therapy.

To test the hypothesis that MEKi treatment engenders an immune contexture and cell state more conducive to immune checkpoint blockade therapy, V_Pr_ tumors were stratified to receive α-PD-L1, cobimetinib, or the combination treatment. Anti-PD-L1 had no effect on V_Pr_ tumor growth; however, both cobimetinib and combination treatment caused regression of all lesions (Fig. 4b). Response rates of the cobimetinib-containing treatment arms were comparable; however, only combination treatment displayed increased CD8 infiltration, significant *MHC-I* upregulation, as well as enhanced *Perforin* expression (Fig. 4c, Extended Data Fig. 7e, f). These data suggest that combination MEKi and α-PD-L1 checkpoint blockade results in a more robust immune activation. Indeed, combination treatment of V_Pr_ tumors generated a durable anti-tumor effect that was maintained in >85% of animals for more than 100 days, doubling overall survival in second-line treatment from 41 to 86 days, relative to MEKi alone (Fig. 4d, e, Extended Data Fig. 8a). Notably, no animals were killed in the combination treatment group due to tumor regrowth. Combination treatment resulted in sustained increases in *CD8*, *IFNγ*, and *Granzyme A*, and significantly increased *MHC-I* expression in all combination-treated samples (Extended Data Fig. 8b–e). Taken together, these results show that combination MEKi and α-PD-L1 checkpoint blockade cooperate to provide a durable anti-tumor response to the unique state created during vemurafenib monotherapy relapse.

Clinical data analyses have illustrated reduced efficacy of both targeted and immunotherapy following BRAFi treatment in melanoma, spotlighting the need for a more complete understanding of how prior lines of therapy can impact responsiveness. Importantly, as combinatorial therapeutic approaches targeting multiple tumor compartments are becoming more standard, not only the malignant cells but all the tumor compartments will need to be considered in rationale treatment design. Here we begin to unravel how therapy-induced adaptation in several tumor compartments can cooperate to mechanistically undermine follow-on treatment responses. Previous work illuminated the role of matrix stiffness in protecting melanoma cell lines from treatment-induced death [15]. More recently, the complex role of matrix-induced cell-fate changes was reported in vitro [50] and in vivo [27] using cell lines. Herein, we combine these concepts by utilizing an unbiased means of tracing multi-compartment evolution of paired biopsies in autochthonous tumors. We discover that our model converges on a distinct tumor cell state, characterized by dedifferentiated tumor cells, a stiffer stromal matrix, and a unique immune contexture. Our work demonstrates that treatment-induced matrix evolution can function to impart cell-fate changes that decrease sensitivity to targeted therapies and further, by reducing antigen presentation machinery and expression, impairs immune-mediated approaches. Moreover, we demonstrate that specific therapeutic interventions can revert many of these features (Extended Data Fig. 9a–d).

The critical importance of previous lines of treatment in determining response to follow-on regimens is traditionally not addressed preclinically and is a key, translatable, finding from our work. By demonstrating fidelity between the resistance phenotype observed in the model and that of greater than half of patient samples from two independent clinical cohorts, we provide the rationale for studying such a prevalent clinical outcome and, moreover, for applying this particular model system to elucidate mechanistic features of this type of therapeutic resistance. Hence, insights from our second-line treatment studies can be leveraged to rationalize cooperativity and durability of an immune-based combination strategy.

## Supplementary information

Supplementary Table 1

Supplemental figure legends

supplemental figure legends

Supplementary Data Set 1
