# Supplementary material for: Therapeutic resistance and susceptibility is shaped by cooperative multi-compartment tumor adaptation
Source: Cell Death Differ. 2019 Mar 1;26(11):2416–29. doi: 10.1038/s41418-019-0310-0 (PMC6889278; doi:10.1038/s41418-019-0310-0)
Supplement: Supplementary file 1 — Supplementary Table 1 [file 41418_2019_310_MOESM1_ESM.pdf]

Mouse ID	Sex	Time Between Initial Biopsy And Progressed Biopsy	Non-Tumor Control Tissue
2295	M	70 days	Muscle
2327	F	119 days	Muscle
2330	F	22 days	Muscle
2352	M	133 days	
2407	F	84 days	Muscle
2413	F	22 days	
2457	M	91 days	
2658	M	35 days	
2659	M	35 days	
2665	M	133 days	Muscle
2746	F	77 days	Muscle
2779	M	126 days	
2786	M	126 days	
2788	F	105 days	
2831	F	126 days	
2846	F	63 days	
2854	F	35 days	
