# Supplementary material for: Therapeutic resistance and susceptibility is shaped by cooperative multi-compartment tumor adaptation
Source: Cell Death Differ. 2019 Mar 1;26(11):2416–29. doi: 10.1038/s41418-019-0310-0 (PMC6889278; doi:10.1038/s41418-019-0310-0)
Supplement: Supplementary file 2 — Supplemental figure legends [file 41418_2019_310_MOESM2_ESM.docx]

**Extended Data Figures**

**Extended Data Figure 1 | *Mitf* expression, initial tumor volume, MAPK activation and genomic copy number changes in relation to vemurafenib-progression.** **a**, Normalized RNA-Seq RPKM initial biopsy values plotted against time to progression for the 17 matched biopsy animals. **b**, Normalized RNA-Seq *Mitf/Axl* RPKM ratios from initial biopsy values plotted against time to progression for the 17 matched biopsy animals. **c**, Initial tumor burden plotted against time to progression for 75 vemurafenib treated animals demonstrating no correlation between *Mitf*, *Mitf/Axl* ratio or initial tumor burden on time to progression. **d**, Quantification of immunohistochemistry staining for phospho-ERK positive cells / total cells, plotted as a percent change normalized to vehicle controls for V_12d_(n=4), Co_12d_(n=4) and V_Pr_(n=5) samples. **e**, Immunohistochemistry staining for tdTomato (red), phospho-ERK (green) and DAPI (blue) in 3 independent vemurafenib-progressed mouse melanoma samples showing phospho-ERK positive, tdTomato negative cells (green arrowheads); phospho-ERK negative, tdTomato positive cells (asterisks) and phospho-ERK positive, tdTomato positive cells (white arrows). **f**, In order to understand the baseline genomic landscape in this model, we performed exome sequencing on tumor biopsies and matched normal tissues (n=6 pairs, Extended Data Table 1). We found an average of 2.25 protein altering mutations (PAMs) per mouse, none of which were in cancer related genes and few copy number alterations (CNAs) (data not shown). This low level of PAMs is consistent with that observed for other MAPK pathway-initiated GEMMs. This figure shows the copy number alterations across all chromosomes for vemurafenib-progressors. **g**, Allelic fraction of Braf^V600E^ comparing the 17 matched IB and V_Pr_ samples. No significant changes were detected in any *Braf* exons in vemurafenib-progressed murine tumors, consistent with the lack of significant change in *Braf* expression (1.5 fold). **h**, Heat map of various RTKs thought to be involved in vemurafenib resistance for each of the 17 matched IB vs. V_Pr_ samples. Values are plotted as %IB value, black boxes indicate >2000% IB value. No statistically significant changes ≥ 2-fold were observed. Mouse IDs are listed above the heat map. IB, initial biopsy; V_Pr_, vemurafenib-progressed samples. Data are plotted as the mean ± SD for **d,g**. Scale bar represents 30µm in **e.** C, control vehicle treated samples; IB, initial biopsy; V_12d_, 12-day vemurafenib treated samples; Co_12d_, 12-day cobimetinib treated; V_Pr_, vemurafenib-progressed samples.

**Extended Data Figure 2 | Immune compartment characterization of vemurafenib progressors. a**, Gene Set Enrichment Analysis (GSEA) results showing normalized enrichment score for indicated gene signatures. **b**, FACS analysis of CD8+ cells in vemurafenib-progressors (n=7). **c**, FACS analysis of CD11b+Gr1+ cells, plotted as a percent of total CD45+ cells from C(n=2) and V_Pr_(n=7) samples. **d**, Percent change from initial biopsy normalized RNA-Seq RPKM values plotted for 17 matched biopsies for *PD-L1* and *PD-L2*. **e**, Immunohistochemical analysis of CD8+ cells / total cells in 12-day vemurafenib treated (n=4) samples. **f**, Immunohistochemical analysis of Gr1+ cells / total cells in 12-day vemurafenib treated (n=4) samples. **g**, Luminex analysis of TNFα expression from C(n=7), V_12d_(n=5) and V_Pr_(n=7) tumors. Data are plotted as the mean ± SD for **b-g**. *p<0.05 by t-test; **p<0.005 by t-test. C, control vehicle treated samples; IB, initial biopsy; V_12d_, 12-day vemurafenib treated samples; V_Pr_, vemurafenib-progressed samples.

**Extended Data Figure 3 | Vemurafenib treatment causes decrease in various melanoma differentiation genes.** **a**, Heat map of normalized RNA-Seq RPKM values for matched pre-treatment and vemurafenib-progressed murine melanoma biopsies (n=17 pairs) using **a**, the 21 gene signature from (Köhler, C. *et al.*, 2017)^5^ and **b**, 8 gene signature from Figure 2a. **c**, Normalized RNA-Seq RPKM values plotted for matched pre-treatment and vemurafenib-progressed murine melanoma biopsies (n=17 pairs). Of note, transcriptional changes in *Mitf* were not prominent (1/17 up 2-fold), but when present, terminal melanocyte differentiation genes were concurrently altered, the two animals that had high *Mitf* levels are highlighted in red and blue. **d**, Differentiation change from baseline score of melanoma marker genes (*Pmel, Lef1, Mlana, Tyrp1, Mc1r, Gpr143*) in matched pre-treatment and 12-day vemurafenib treated biopsies (n=5 pairs) and **e**, in matched pre-treatment and vemurafenib-progressed biopsies (n=17 pairs). Dark blue bar indicates animal that had high *Mitf* expression (#2327). IB, initial biopsy; V_Pr_, vemurafenib-progressed samples.

**Extended Data Figure 4 | Expression of melanoma differentiation genes in patient samples. a**, Differentiation change from baseline score of melanoma differentiation genes from Figure 2a (*EDNRB, PMEL, LEF1, MLANA, TYRP1*) in matched pre-treatment and vemurafenib-progressed biopsies from melanoma patient samples (n=23 pairs) and **b**, in matched pre-treatment and BRAFi-progressed biopsies from melanoma patient samples from (Hugo, W. *et al.*, 2015)^20^ (n=31 pairs). **c**, Differentiation change from baseline score of murine-derived melanoma differentiation signature (*BACE2, CITED1, DCT, EDNRB, GPNMB, GPR143, KIT, MC1R, MITF, MLANA, OCA2, PAX3, PMEL, RARB, SLC24A4, SLC24A5, SLC45A2, TRPM1, TYR, TYRP1,* *ZEB2*) from (Köhler, C. *et al.*, 2017)^5^ in matched pre-treatment and BRAFi-progressed biopsies from melanoma patients from (Hugo, W. *et al.*, 2015)^20^ (n=31 pairs). **d**, Normalized Nanostring CPM initial biopsy values for *MITF* plotted against time to progression for 20 patients. **e**, Normalized *MITF/AXL* Nanostring CPM ratios from initial biopsy values plotted against time to progression.

**Extended Data Figure 5 | Single cell RNA-Seq characterization. a**, Heatmap showing average expression (z-score transformed) of select marker genes (rows) across the different cell clusters (columns) in the single cell RNA sequencing data set. Yellow indicates high, pink low expression. **b**, Bar graph showing the fraction of immune cells being categorized as either monoctyes/macrophages (Mono/Macro), NK, T or NKT cells (NK/T cells), neutrophils or mast cells in control, V_12d_ and V_Pr_ tumors. **c**, Single cell RNA-Seq violin plots showing the expression in non-immune stromal cells of *Tgfb1*, *Col1a1*, the collagen cross-linking enzyme *Tgm2* and a fibroblast TGFβ response signature (F-TBRS) split by treatment. Number of cells per group is given below the violin plots. * p<0.05, ** p<0.01, **** p<0.0001 by Wilcoxon rank sum test in **c**. C, control vehicle treated samples; V_12d_, 12-day vemurafenib treated samples; V_Pr_, vemurafenib-progressed samples.

**Extended Data Figure 6 | Vemurafenib-associated matrix remodeling impacts melanoma cell state changes.** **a**, Images showing both human and mouse untreated melanoma samples (upper) and vemurafenib-progressed samples (lower). **b**,**c** Mass spectrometry of pyridinoline (PYD) and deoxypyridinoline (DPD) normalized to total collagen from C (n=26) and V_Pr_ (n=26) tumors. **d**, Gene expression analyses of A375 cells 36 hours after plating in varying stiffness [0.2kPa (n=3), 3kPa (n=3), 12kPa (n=3)] of polyacrylamide/bis-acrylamide gels. Data is plotted as normalized to average 0.2kPa conditions. **e**, Differentiation change from baseline score of melanoma differentiation signature (*ENDRB, S100A1, PMEL, LEF1, MLANA, TYRP1, MC1R, GPR143*) of WM266-4 cells 36 hours after plating in varying stiffness [0.2kPa (n=3), 3kPa (n=3), 12kPa (n=3)] of polyacrylamide/bis-acrylamide gels. **f**, Gene expression analyses of WM266-4 cells 36 hours after plating in varying stiffness [0.2kPa (n=3), 3kPa (n=3), 12kPa (n=3)] of polyacrylamide/bis-acrylamide gels. Data is plotted as normalized to average 0.2kPa conditions. **g**,**h,** Colo829 cells 27.75 hours after plating in varying stiffness [0.2kPa (n=3), 3kPa (n=3)] of polyacrylamide/bis-acrylamide gels and incubated with CytoTox Red reagent to assess dead cells, plotted as counts per mm^2^ in **g**, and total counts in **h**. C, control vehicle treated samples; V_Pr_, vemurafenib-progressed samples. Data are plotted as the mean ± SD for **b,c,d**. Scale bar represents 100 µm in **a**. * p<0.05, ** p<0.005, *** p<0.0005, **** p<0.00005 by t-test. C, control vehicle treated tumors; V_Pr_, vemurafenib-progressed samples.

**Extended Data Figure 7 | Cobimetinib treatment after vemurafenib progression reveals altered gene expression and genomic changes. a**, Progression free survival plot of cobimetinib treatment in previously untreated tumors (blue, n=14; Cobimetinib 1^st^ Line) and in vemurafenib-progressed tumors (orange, n=6; Cobimetinib 2^nd^ Line). Animals were classified as progressed when their tumor burden reached any growth above their initial biopsy value (cobimetinib 1^st^ line; blue) or crossover biopsy value (cobimetinib 2^nd^ line; orange). **b**, Sum of normalized 2^-dCt^ values of MAPK target genes plotted as percent of V_Pr_ for the 7-day 2^nd^ line cobimetinib treatment (n=4). **c**, Copy number alterations across all chromosomes for cobimetinib 1^st^ line progressors. **d**, Immunohistochemistry quantification of Gr1+ cells / total cells plotted for V_Pr_(n=8) and Co(n=4) tumors. **e**, Immunohistochemistry quantification of CD8+ cells / tissue area plotted for V_Pr_(n=2), P(n=6), Co(n=4) and CP(n=4) tumors. **f**, Fluidigm analysis of *H2-k* transcript plotted as a percent of V_Pr_ plotted for P(n=3), Co(n=4) and CP(n=4) tumors. Data are plotted as the mean ± SD for **d,e.** P values in **a** by log-rank (Mantel-Cox) test. *p<0.05 by t-test; **p<0.005 by t-test. 7d PD, vemurafenib-progressed samples that are treated for 7 days with various 2^nd^ line treatments; Co, vemurafenib-progressed cobimetinib treated samples; CP, vemurafenib-progressed cobimetinib and α-PD-L1 treated samples; IB, initial biopsy; P, vemurafenib-progressed α-PD-L1 treated samples; V_Pr_, vemurafenib-progressed samples.

**Extended Data Figure 8 |** Combination treatment after vemurafenib progression shows differential effects compared to cobimetinib alone. **a**, Tumor volume growth plot expressed as a percentage of crossover volume for vemurafenib-progressed animals crossed over to 2^nd^ line treatment for α-PD-L1 (P, gray, n=6), cobimetinib (Co, orange, n=6) or cobimetinib + α-PD-L1 (CP, purple, n=7) animals. **b-e**, Expression of the indicated genes in terminal tumors from **a** plotted as percent *SP2* normalized 2^-dCt^ values for Co (n=3) and CP (n=4) samples. **p<0.005 by t-test. EOS, end of study tumors from vemurafenib-progressed 2^nd^ line treatments as listed; V_Pr_, vemurafenib-progressed samples.

**Extended Data Figure 9 | Therapy-induced stromal remodeling characterizes evolution of resistance in *Braf* mutant melanoma and impacts treatment responsiveness.** **a**, Therapeutic relapse via targeted BRAF inhibition (vemurafenib) was accompanied by tumor cell fate cell fate conversion, increased matrix stiffness and changes in the tumor immune contexture. Features that demonstrate fidelity with patient tumors. Despite the lack of evidence supporting a common genetic mechanism to explain the relapse, tumor cell phenotypes converged on a unified mechanism of reduced melanocyte cell fate, which was shown *ex vivo* to be mediated solely through matrix-mediated changes. **b**, Prior treatment with BRAFi significantly altered the response and durability to MEKi treatment (naïve vs 1^st^ line relapse setting). **c**, Further evaluation of MEKi treatment impact on the relapsed state demonstrated changes in tumor cell fate and immune contexture **d**, that could favorably combine with immune checkpoint inhibition *in vivo*. Notably, relapse with MEKi treatments both resulted in resistance accompanied by the acquisition of genetic alterations, indicating that distinct selective pressures mediated divergent paths to relapse.
