# Supplementary material for: Therapeutic resistance and susceptibility is shaped by cooperative multi-compartment tumor adaptation
Source: Cell Death Differ. 2019 Mar 1;26(11):2416–29. doi: 10.1038/s41418-019-0310-0 (PMC6889278; doi:10.1038/s41418-019-0310-0)
Supplement: Supplementary file 3 — supplemental figure legends [file 41418_2019_310_MOESM3_ESM.pdf]

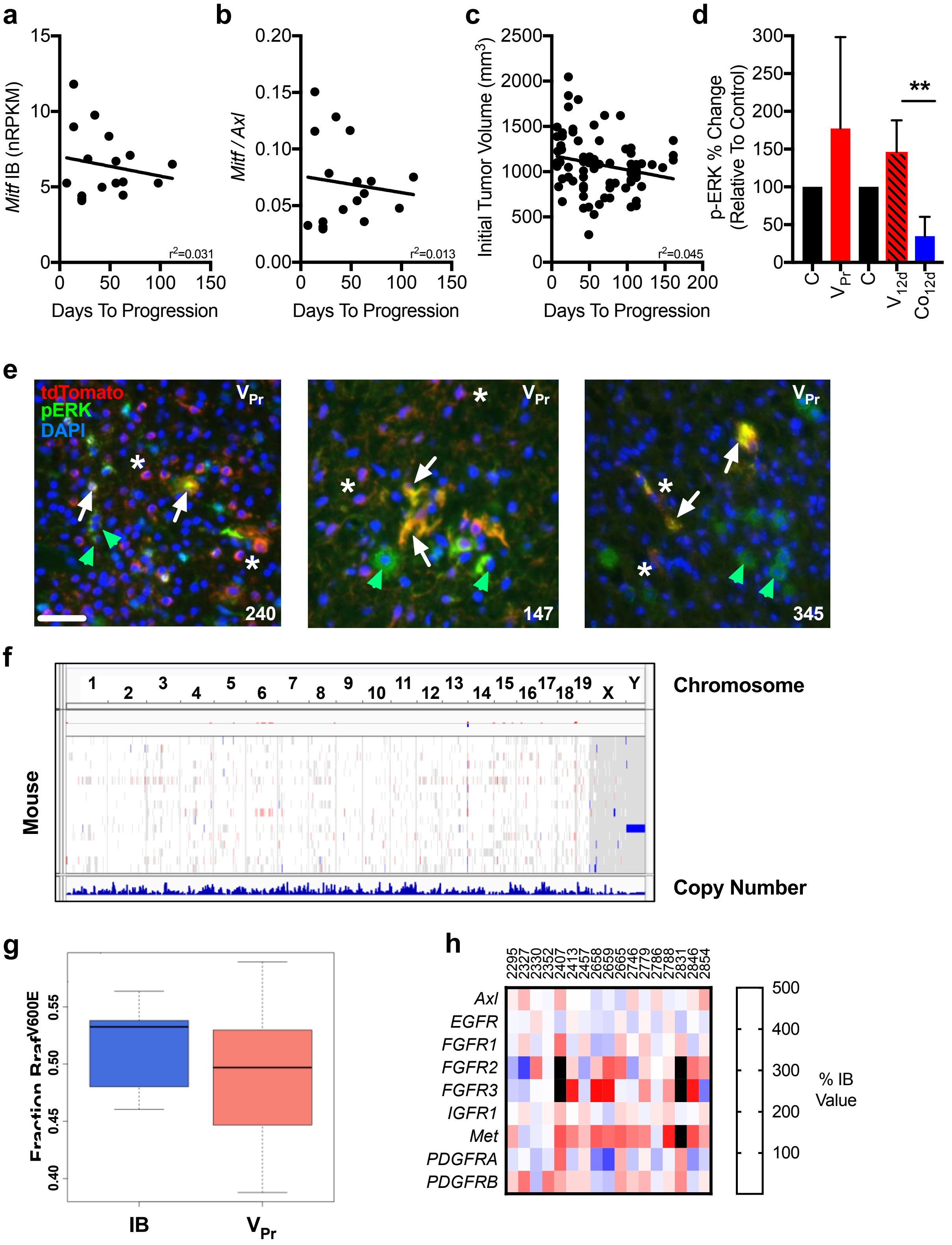

Extended Data Figure 1

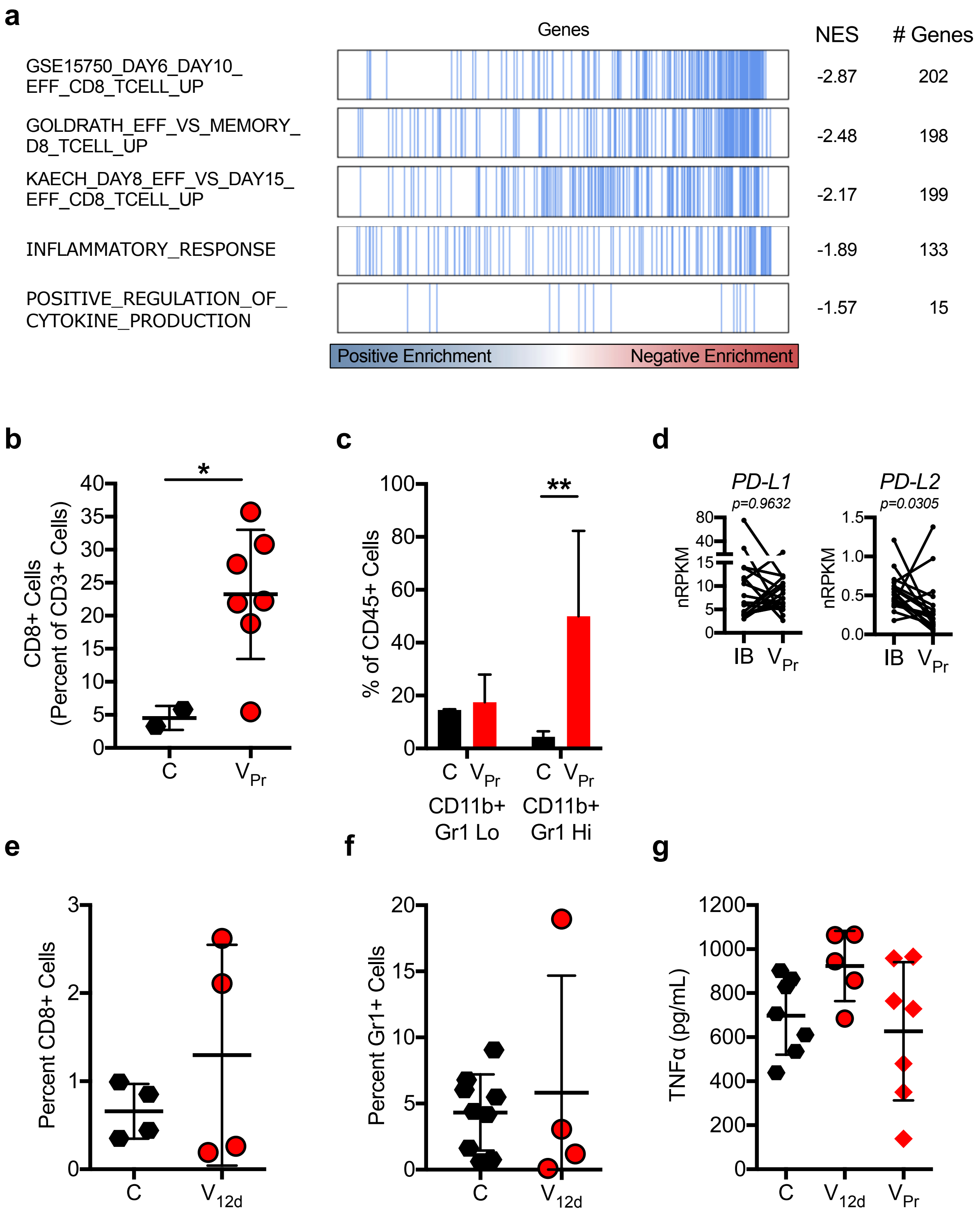

Extended Data Figure 2

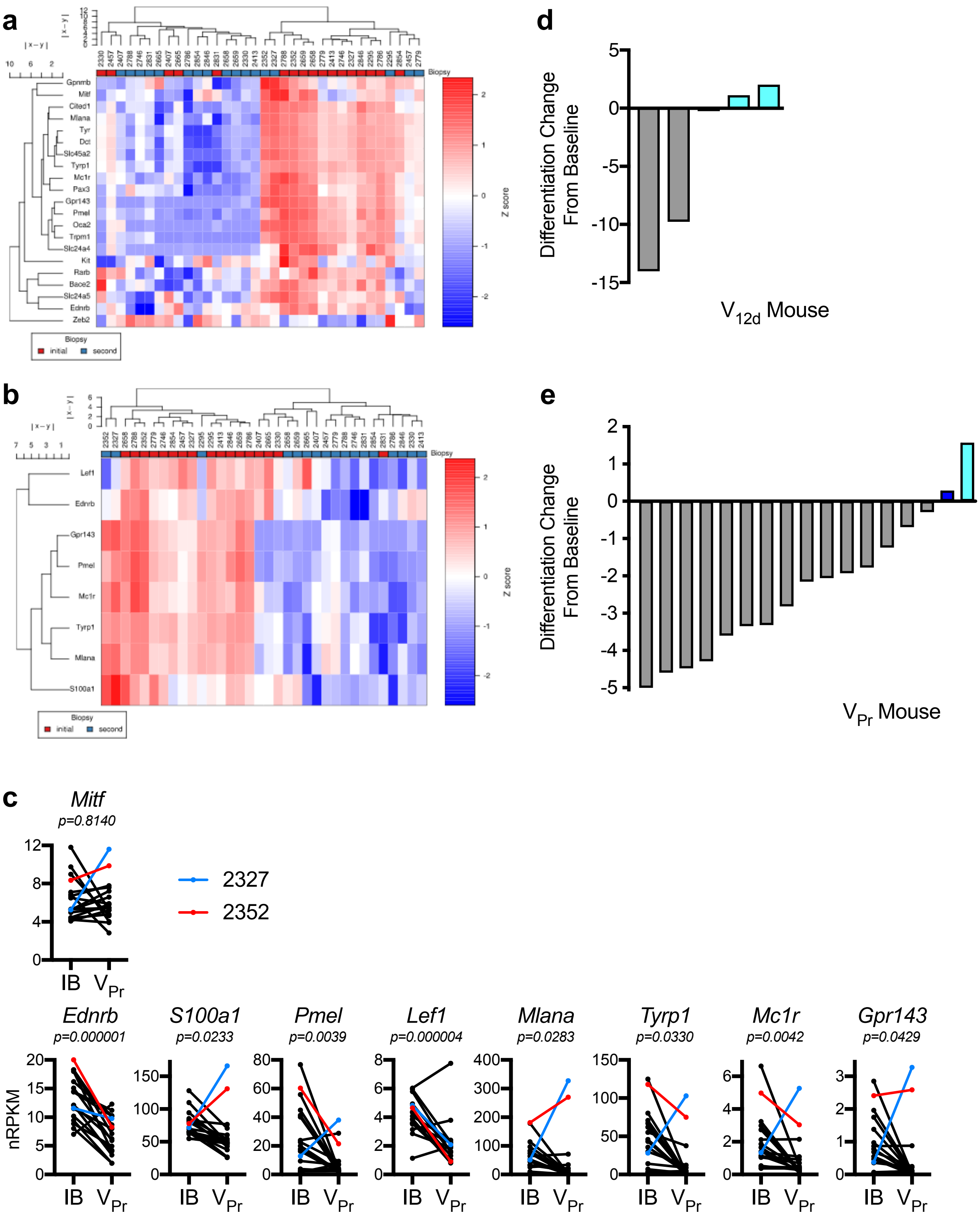

Extended Data Figure 3

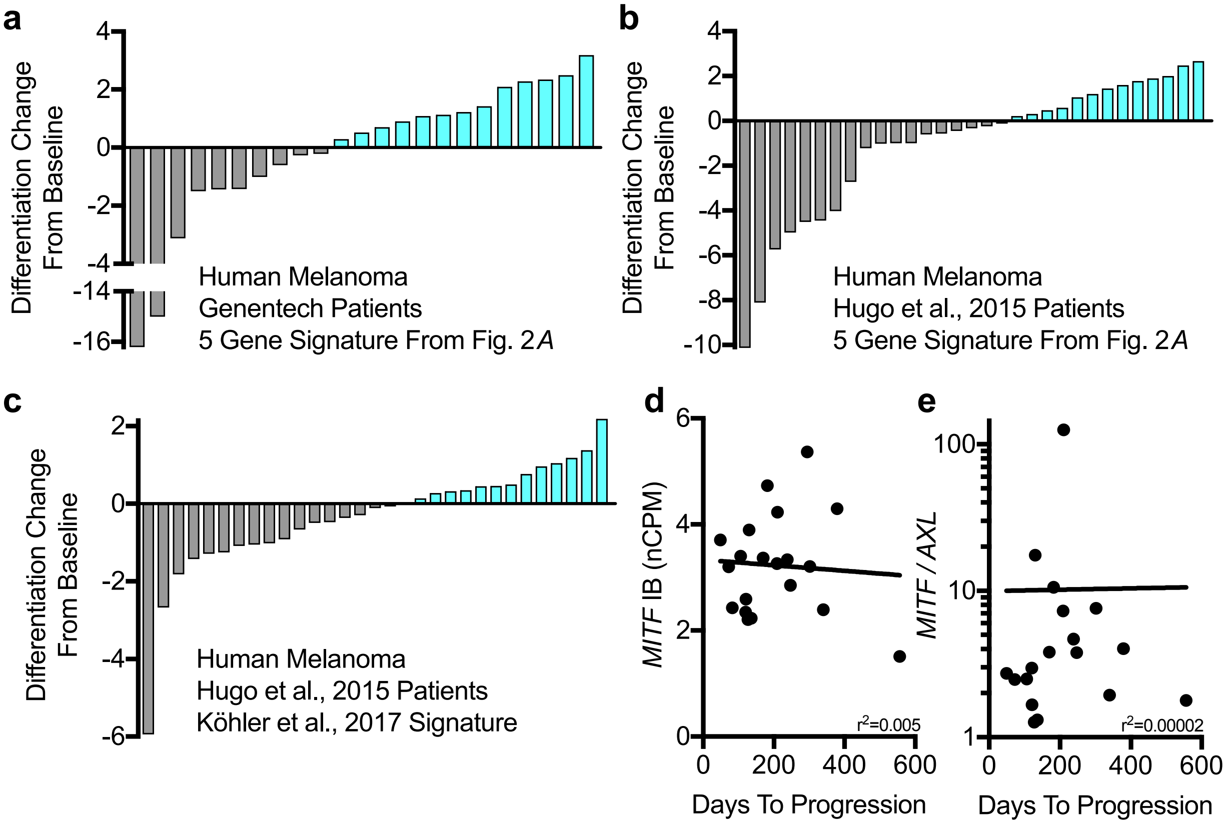

Extended Data Figure 4

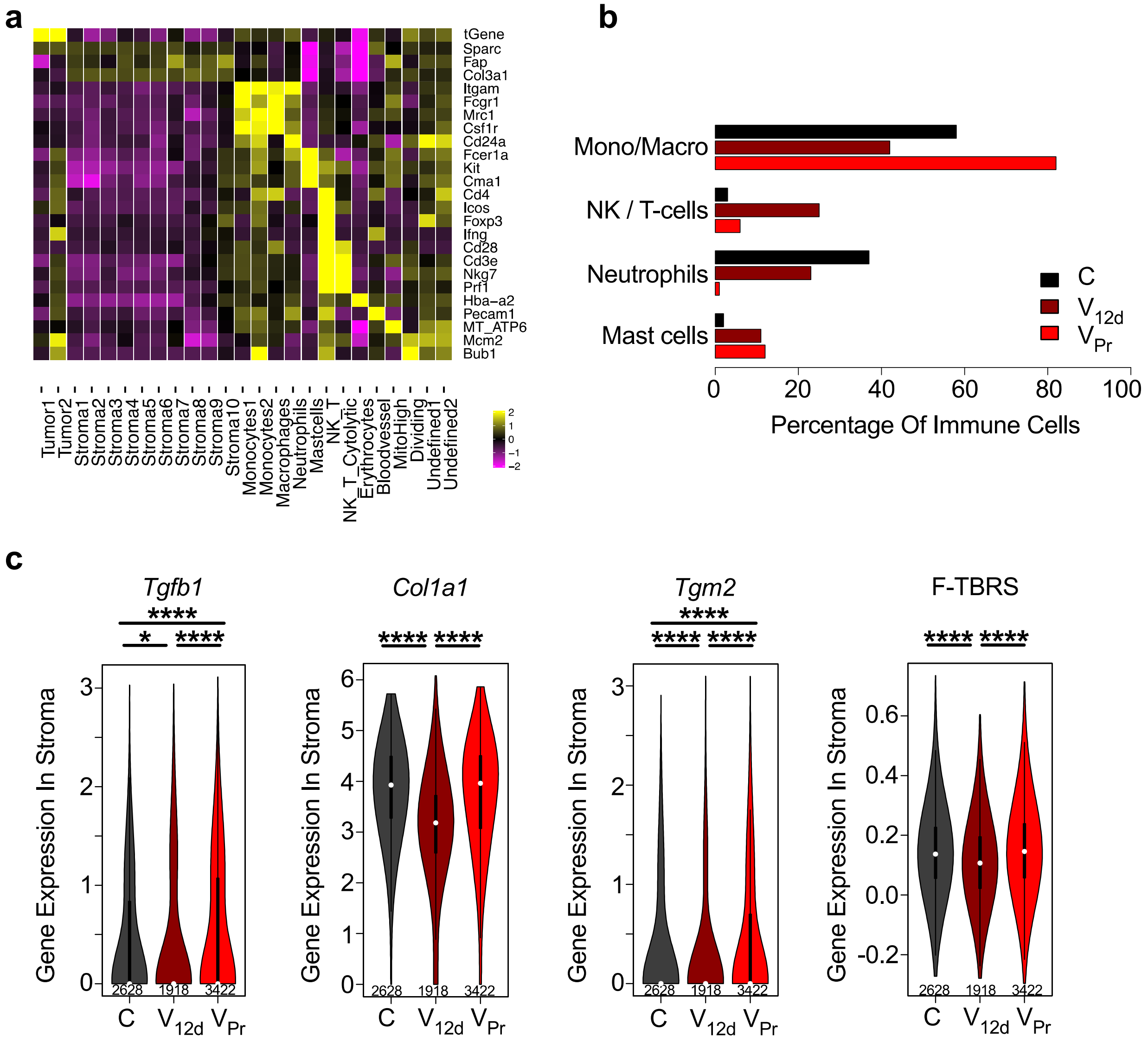

Extended Data Figure 5

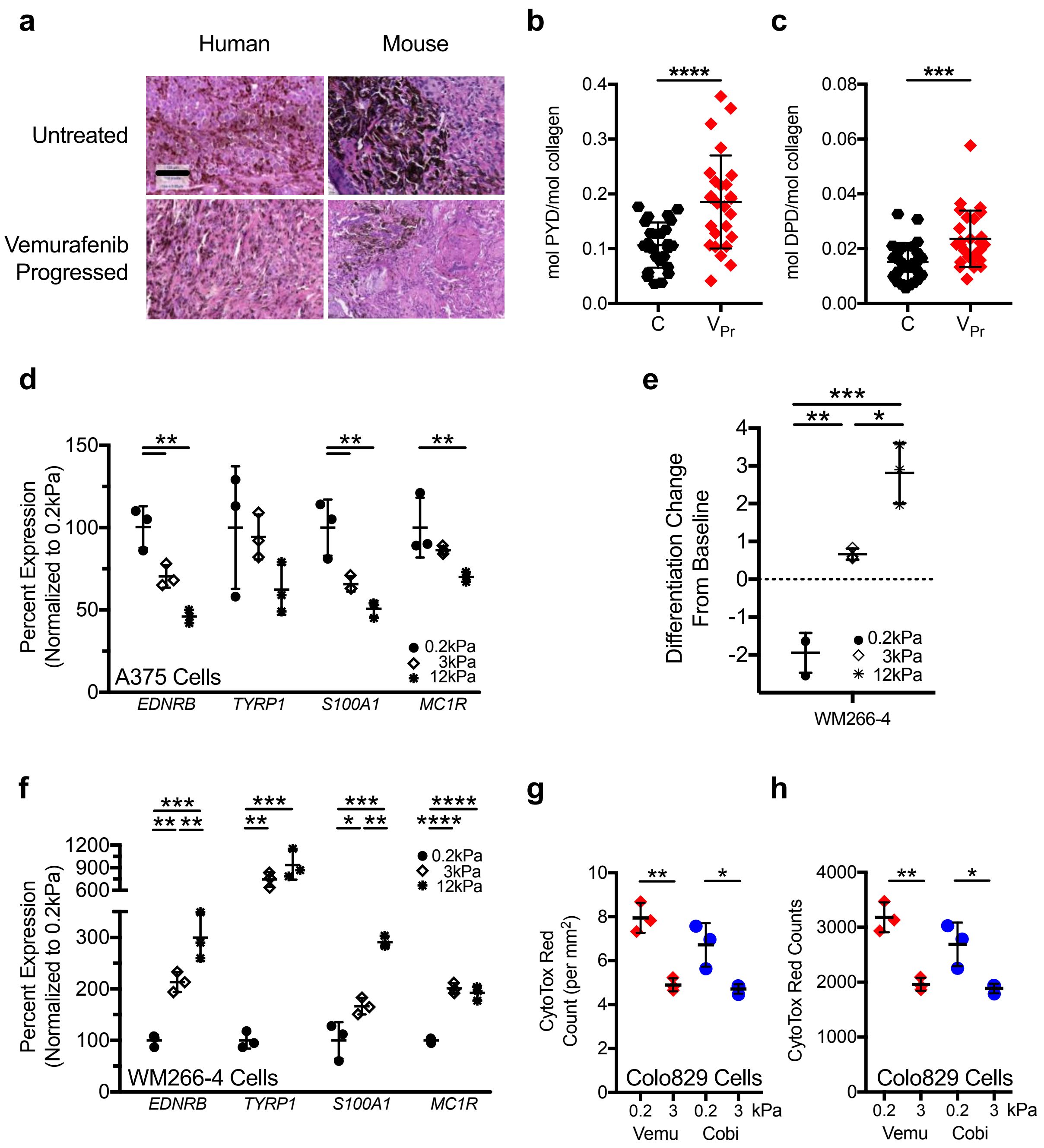

Extended Data Figure 6

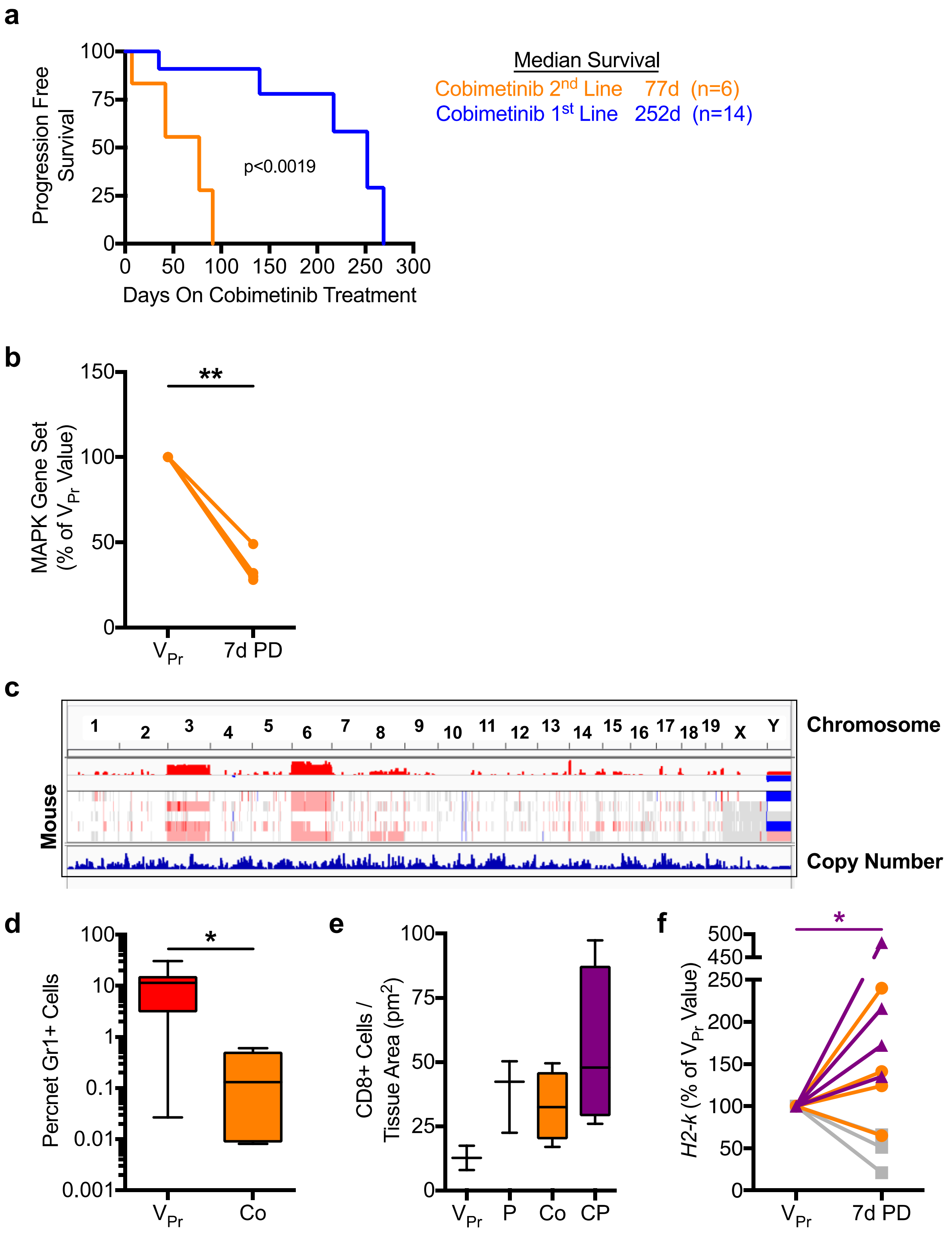

Extended Data Figure 7

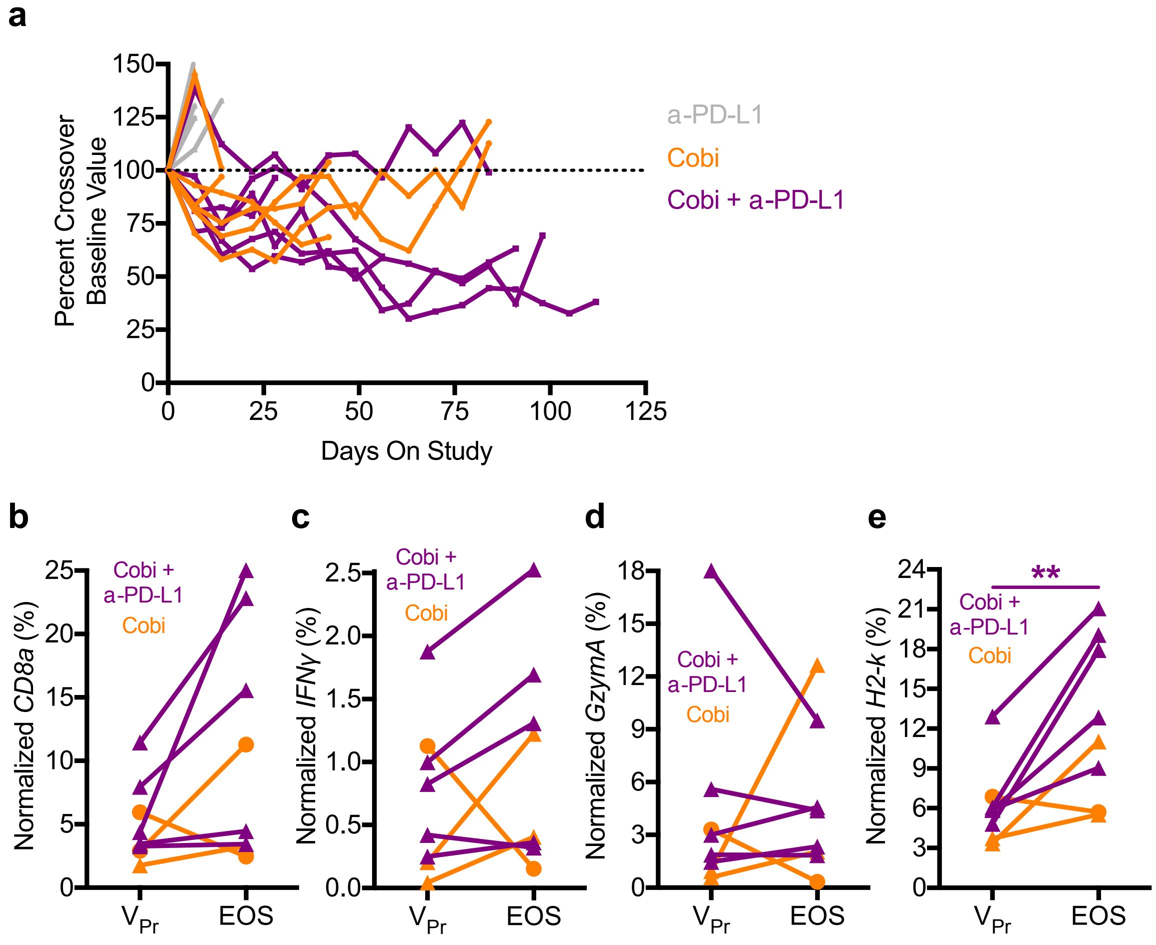

Extended Data Figure 8

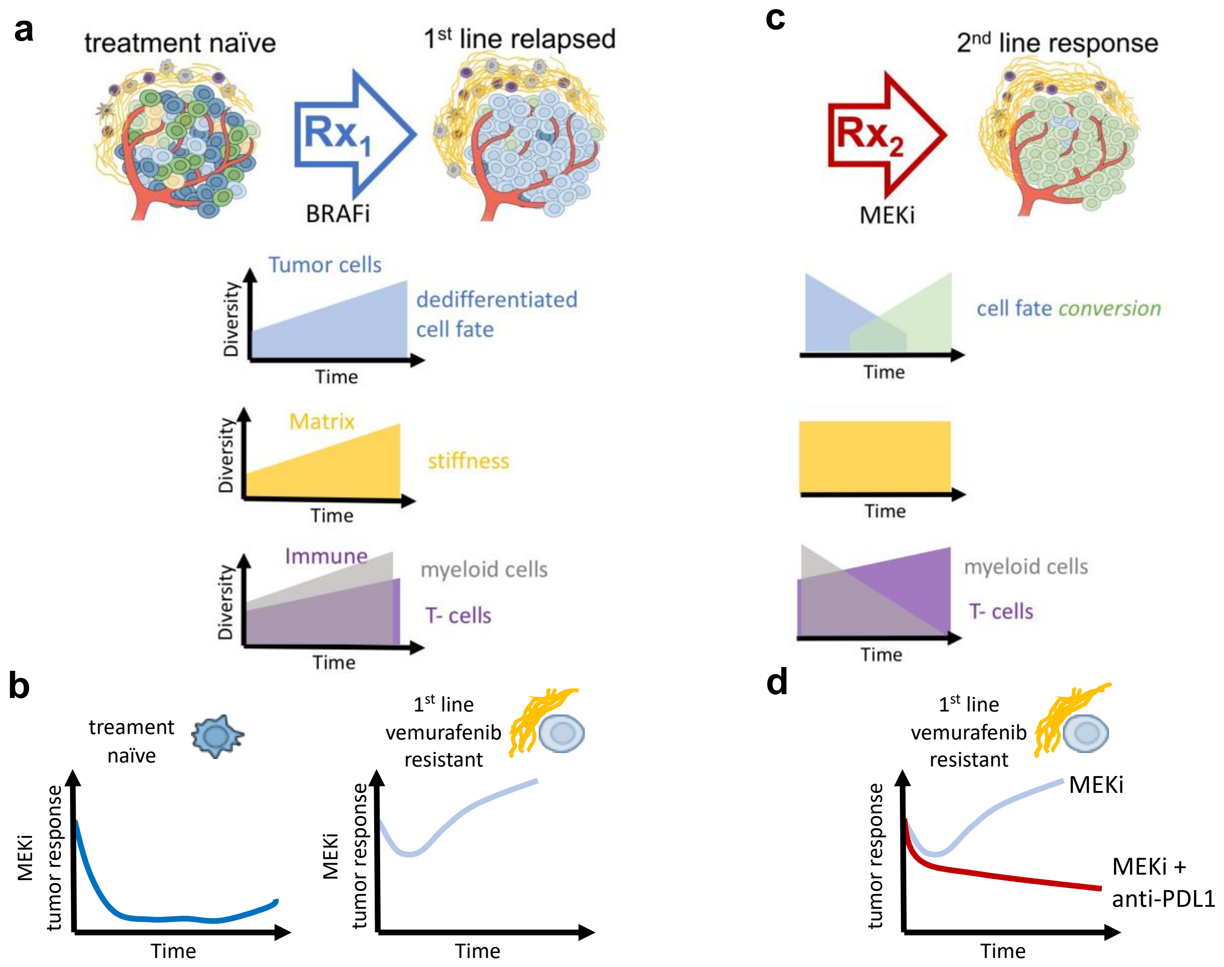

Extended Data Figure 9
